# Genetic diversity analysis of *Iris germanica* cultivars based on ISSR and SRAP molecular markers

**DOI:** 10.3389/fpls.2025.1629234

**Published:** 2025-09-10

**Authors:** Wei Bo, Song Wang, Guoming Xing, Yuguo Wang

**Affiliations:** ^1^ College of Horticulture, Shanxi Agricultural University, Taigu, Shanxi, China; ^2^ College of Agriculture, Shanxi Agricultural University, Taigu, Shanxi, China

**Keywords:** *Iris germanica*, genetic diversity, ISSR, SRAP, clustering analysis

## Abstract

**Introduction:**

*Iris germanica* L. (1753), commonly known as bearded iris, is a popular ornamental plant species appreciated for its beautiful and diverse flower colors and forms. Despite its aesthetic appeal, there is limited knowledge about the genetic diversity and relationships among *Iris germanica* cultivars.

**Methods:**

To address this, in this study, we investigated the genetic diversity and molecular clustering of *Iris germanica* cultivars through ISSR and SRAP molecular markers.

**Results:**

Our analysis identified that ISSR analysis revealed a high level of genetic diversity among *Iris germanica* cultivars. The 9 ISSR primers generated 72 amplified bands, of which 66 were polymorphic, yielding a polymorphism percentage of 89.86%. Similarly, SRAP analysis demonstrated substantial intraspecific genetic diversity. 12 SRAP primer pairs produced 693 amplified bands, with 669 exhibiting polymorphisms, corresponding to a polymorphism rate of 96.54%. Genetic similarity coefficients ranged from 0.513 to 0.868 for ISSR and 0.595 to 0.801 for SRAP markers, highlighting variations and relationships among *Iris germanica* accessions. ISSR Molecular marker cluster analysis categorized divides *Iris germanica* cultivars with similar morphological characteristics into distinct groups to a certain extent based on genetic similarity coefficients, and SRAP marker could also make the same species from different regions first cluster into one group based on classifying the similar phenotypic *Iris germanica* varieties, indicating that the SRAP marker used to analyze the genetic diversity of *Iris germanica* cultivars were more accurate than the ISSR marker. Combining both ISSR and SRAP markers not only effectively distinguished between dwarf and tall species of *Iris germanica*, but also separately isolated two *Iris germanica* species from Shanxi province. It had also been found that *Iris germanica* Antique Red, *Iris germanica* Indian leader were clustered into one group and *Iris germanica* Bloodstone and Cherry Garden were gathered together in three kinds of clustering methods, indicating that these varieties had very close relationship.

**Discussion:**

Overall, this study provides valuable insights into the genetic diversity and relationships within Iris species, offering important implications for breeding and conservation efforts.

## 1 Introduction

As the pace of urbanization accelerates worldwide, people increasingly recognize the importance of greenery for a healthy life (Ridder et al., 2004). However, the hard paving of numerous urban buildings and roads in the ecological environment has replaced natural land and some plants, as well as brought about a series of negative impacts such as environmental pollution, making the environmental space for human survival deteriorate day by day. Subgenus *Barbellae* (genus *Iris*), belonging to the Iridaceae family, is a perennial herbaceous plant and is one of the worlds famous rhizomatous flowers (Bo et al., 2017). With a variety of plant species, diverse flower colors, and unique flower shapes, it serves not only as an ideal ground cover plant for urban greening but also plays a significant role in improving the urban ecological environment and maintaining ecological balance (Zhang and Chen, 2013).

In recent years, the continuous improvement in peoples quality of life has also led to an increasing demand for irises in both domestic and international markets, and higher requirements for their ornamental and ecological values have been set. *I. germanica*, a representative species of this Subgenus *Barbellae*, has been widely used in garden landscaping due to its rich flower colors, numerous varieties, and large flower size (Yang et al., 2021). Relevant studies have shown that German iris varieties have a high rate of cross-pollination, coupled with their strong value in garden utilization, research on their introduction, cultivation, and hybrid breeding is the most extensive (Ji, 2013). A variety of cultivated and mutant varieties have emerged, with high variability, and long-term intraspecific hybridization has also made the genetic background of German iris varieties more complex.

Currently, domestic and international research on the biological characteristics (Zhang et al., 2015), stress resistance (Bo et al., 2017; Van Zandt et al., 2003; Wang et al., 2001; Liu et al., 2021), reproductive biology (Zhang et al., 2025; Sandenbergh et al., 2025), cut flowers (Yu et al., 2017), chromosome karyotype (Choi et al., 2020; Park et al., 2022), and biochemistry (Yu et al., 2017; Chandni et al., 2024) of Iris plants has been actively carried out. As molecular techniques for studying plant genetic diversity, DNA molecular markers and sequence analysis, with their advantages of being efficient, safe, reliable, and convenient, have been widely used in analyzing plant genetic diversity (Liu et al., 2002; Ibrahimi et al., 2024; Ghonaim et al., 2024; Li et al., 2024). ISSR (Inter-Simple Sequence Repeat) and SRAP (Sequence-Related Amplified Polymorphism) are powerful molecular marker techniques, each with its own set of advantages that make them indispensable in genetic research. ISSR is favored for its ease of use, cost-effectiveness, high polymorphism, rapid results, broad applicability, and consistent repeatability, allowing for straightforward and economical experiments without prior DNA sequence knowledge. It generates highly polymorphic DNA fingerprints that effectively distinguish between genetic individuals (Yao et al., 2005; Gemmill and Grierson, 2021; Sevindik et al., 2023). SRAP, in contrast, is celebrated for its high polymorphism, resolution, stability, wide applicability, automation-friendly nature, and the convenience of not needing specific sequence information. It excels at revealing minute genetic variations, making it a robust tool for detailed genetic mapping. The combination of these techniques offers researchers a comprehensive toolkit for genetic diversity analysis, germplasm evaluation, and high-throughput screening, enhancing the depth and breadth of genetic information obtained (Li et al., 2007; Li et al., 2013).

In recent years, the use of these molecular markers in systematic taxonomy, variety identification, and kinship analysis of Iris plants has also been reported, gradually becoming a new focus of modern Iris research (Weber et al., 2020; Nikitina et al., 2023). Sun Mingzhou (Sun, 2012) developed microsatellite primers in *Iris laevigata* Fisch., and used these primers for the study of genetic structure in Iris plants, showing that most of the developed microsatellite primers obtained the target fragments among eight closely related species in the Iris genus, which can meet the requirements for analyzing genetic structure. Makarevitch et al. (2003) used the non-coding intergenic spacer sequences of the *trnL-F* and *trnL* regions and 56 RAPD markers to construct two phylogenetic trees for 22 Iris plants distributed in the Siberian region, reconstructing the taxonomic system of *Iris* plants in the Siberian region. In studies utilizing ISSR and SRAP markers for genetic analysis of *Iris* species, significant progress has been made in understanding population genetics and phylogenetic relationships (Du, 2008). Tong et al. (2019) conducted a genetic characterization and phylogenetic analysis of 40 *Iris* germplasm resources using SRAP molecular markers. From an initial screening of 170 random SRAP primers, 10 primers were selected based on their clear banding patterns, high polymorphism, good reproducibility, and strong stability. These primers collectively detected 168 loci, all of which were polymorphic, resulting in a polymorphism rate of 100%. Similarly, Zhang et al. (2007) employed RAPD and ISSR markers to examine four wild Iris species from different regions, revealing significant interspecific genetic variation that exceeded intraspecific differences. Tong et al. (2015) investigated the genetic characteristics and phylogenetic relationships of 34 *Iris* horticultural cultivars using ISSR markers. The analysis revealed a 100% polymorphism rate in the amplified bands, demonstrating substantial genetic polymorphism among the tested materials. However, there are fewer reports on the molecular biological systematic evaluation of German iris as a horticultural ornamental variety. The genetic diversity analysis of German iris germplasm is the foundation for deeply exploring excellent varieties, innovation, and seedling work, and is also a prerequisite for the effective application of excellent German iris varieties in urban greening. Fully developing and utilizing Chinas excellent German iris cultivation varieties, accelerating the breeding of high-quality new varieties, is the main direction of future research on Iris germplasm resources.

This study selected 26 German iris varieties from five different regions of China as experimental materials. Using ISSR and SRAP molecular markers, it quantified the genetic diversity of cultivated German iris varieties at the molecular level, and analyzed the kinship among these varieties. The results provide a certain reference basis for further germplasm collection, variety identification, fingerprint map construction, improvement breeding, resource protection and utilization of Iris plants.

## 2 Materials and methods

### 2.1 Experimental materials

The study utilized 26 different varieties of German iris as the subjects of the experiment. All varieties were cultivated in the botanical garden resource nursery at the Institute of Horticultural Research, Shanxi Academy of Agricultural Sciences in 2013, as detailed in **Table 1**.

**Table 1:** Twenty six varieties of experimental plant materials of *Iris germanica*.

Code	Latin names	Origin
1	*Iris germanica* Purple Flower	Shanxi Province
2	*Iris germanica* Tawny	Shanxi Province
3	*Iris germanica* Thrilling	Beijing
4	*Iris germanica* Sauce Yellow	Beijing
5	*Iris germanica* Antique Red	Beijing
6	*Iris germanica* Nautical Flag	Liaoning Province
7	*Iris germanica* Golden Doll	Liaoning Province
8	*Iris germanica* Immortal White	Henan Province
9	*Iris germanica* Dwarf Dream	Liaoning Province
10	*Iris germanica* Music Bor	Beijing
11	*Iris germanica* Purple Glow	Beijing
12	*Iris germanica* Black Flag	Beijing
13	*Iris germanica* Blood stone	Liaoning Province
14	*Iris germanica* White and Yellow	Beijing
15	*Iris germanica* White Calyx	Beijing
16	*Iris germanica* Cherry Garden	Hebei Province
17	*Iris germanica* Ussuri	Hebei Province
18	*Iris germanica* Purple Brown	Hebei Province
19	*Iris germanica* Indian leader	Hebei Province
20	*Iris germanica* Golden Doll	Hebei Province
21	*Iris germanica* Immortal White	Hebei Province
22	*Iris germanica* Dwarf Dream	Hebei Province
23	*Iris germanica* Music Bor	Hebei Province
24	*Iris germanica* Flute Sound	Hebei Province
25	*Iris germanica* Blood stone	Hebei Province
26	*Iris germanica* Antique Red	Hebei Province

### 2.2 Experimental methods

#### 2.2.1 DNA extraction

The DNA extraction process was carried out using an optimized CTAB method. The CTAB extraction buffer consisted of 2% (w/v) CTAB, 100 mM Tris-HCl (pH 8.0), 20 mM EDTA (pH 8.0), 1.4 M NaCl, supplemented with 2% (w/v) PVP-40 (polyvinylpyrrolidone-40) and 0.1% (v/v) β-mercaptoethanol (added freshly before use) (Doyle and Doyle, 1987). Each sample started with 0.5 grams of fresh leaf tissue. All samples were processed individually without pooling. Fully expanded healthy leaves at 30 days post-anthesis (DPA) were harvested between 09:00-11:00 AM, immediately submerged in liquid nitrogen and stored at -80 °C. Specifically, the procedure began by preheating the CTAB solution to 65 °C in a water bath. The plant material was then quickly ground in liquid nitrogen and transferred to a 2 mL centrifuge tube, followed by the addition of 1.5 ml the preheated CTAB solution. The mixture was kept at 65 °C for 30 to 60 minutes, with gentle inversion every 10 minutes to ensure thorough mixing. After an initial centrifugation step at 11000rpm for 5 minutes, the supernatant was carefully transferred to a new tube. An equal volume (1.5ml) of phenol/chloroform (1:1) was added, the mixture was thoroughly blended, and then centrifuged at 11000rpm for another 10 minutes. The supernatant was subsequently moved to a fresh tube. Chloroform was added in an equal volume (1.5ml), mixed well, and centrifuged at 11000rpm for 10 minutes. The supernatant was then transferred to a new tube. Steps 4 and 5 were repeated to ensure thorough purification. Isopropanol, two-thirds the volume of the supernatant, was added to induce DNA precipitation. The mixture was left to stand at room temperature for 15 minutes. After centrifugation at 11000rpm for 6 minutes, the supernatant was carefully discarded. The precipitated DNA was rinsed with 70% ethanol, followed by a centrifugation step at room temperature at 11000rpm for 2 minutes. The supernatant was discarded, and the rinsing step was repeated once more. Finally, for concentration and quality assessment, 2-3μL of the extracted DNA was subjected to agarose gel electrophoresis on a 2.0% gel with TAE buffer and assessed using a NanoDrop 2000 spectrophotometer (Thermo Fisher Scientific, Waltham, MA, USA). Spectrophotometric analysis revealed A260/A280 ratios ranging from 1.82 to 1.95 across all samples, indicating high-purity DNA with minimal protein contamination. Electrophoretic analysis demonstrated sharp and distinct bands without smearing or tailing, confirming the integrity of genomic DNA. The remaining DNA was stored at -20 °C for future applications.

#### 2.2.2 ISSR analysis

Forty ISSR primers were initially considered, from which 9 were selected based on their clear amplification, abundant bands, and high level of polymorphism for use in subsequent experiments (**Supplementary Table S1**). The PCR amplification mixture, with a total volume of 20 μL, 20μL, included 2 μL of DNA template, 2 μL of 10X concentrated buffer, 0.3 μL of primer, 0.4 μL of dNTPs, 0.5 μL of MgCl_2_, 0.2 μL of Taq DNA polymerase, and 14.6 μL of ddH_2_O. The PCR protocol was set with an initial denaturation at 94°C for 5 minutes, then 35 cycles of denaturation at 94°C for 45 seconds, annealing at temperatures ranging from 53 to 62 °C for 45 seconds, and extension at 72 °C for 1.5 minutes, with a final extension at 72 °C for 10 minutes, and the samples were stored at 4 °C. Amplified DNA fragments were visualized and documented using 2% agarose gel electrophoresis and a gel imaging system (Bio-Rad) with Image Lab™ software (v6.1).

#### 2.2.3 SRAP analysis

A total of 154 pairs of SRAP primers were randomly selected (as shown in **Supplementary Table S2**) and 12 pairs were chosen based on their clear background, abundant bands, and high polymorphism for subsequent experiments. The PCR amplification system (20 μL) included 2 μL of DNA template, 1 μL of the upstream primer, 1 μL of the downstream primer, 2 μL of 10X buffer, 0.4 μL of dNTPs, 0.5 μL of MgCl_2_, 0.3 μL of Taq DNA polymerase, and 12.8 μL of ddH_2_O. The PCR amplification program started with an initial denaturation at 94 °C for 3 minutes, followed by 40 cycles of denaturation at 94 °C for 30 seconds, annealing at 36 °C for 50 seconds, and extension at 72 °C for 1.5 minutes, with a final extension at 72 °C for 10 minutes, then stored at 4 °C. Gel electrophoresis using polyacrylamide gel was performed, and images were captured with a gel documentation system for recording.

### 2.3 Data analysis

Binary values of 0 and 1 were assigned to the PCR amplification products from ISSR and SRAP, with the presence or absence of DNA bands at specific gel positions represented as 1 or 0, respectively. Shannons information index (*I*) and Neis gene diversity (*H*) were computed using PopGen32 v1.31. The polymorphism information content (PIC) of ISSR and SRAP markers was determined with PIC_CALC software. The data were processed using NTsys2.10e software (Cheng et al., 2018). The genetic similarity coefficient (Dice) matrix was computed in the “similarity analysis module” from the 0/1 data using the simqual option. Subsequently, the SAHN method was applied in the clustering module for UPGMA cluster analysis (Zhou et al., 2015), resulting in the generation of a dendrogram and a three-dimensional principal component plot based on the genetic matrix. The cophenetic correlation coefficient (CCC) was calculated using the cophenetic module to validate the agreement between the UPGMA dendrogram and the similarity matrix. The CCC value >0.80 was considered indicative of an excellent fit (Sokal and Rohlf, 1962). Analysis of molecular variance was (AMOVA) performed with GenAlEx 6.5 software.

## 3 Results and analysis

### 3.1 ISSR amplification polymorphism analysis

**Figure 1:**
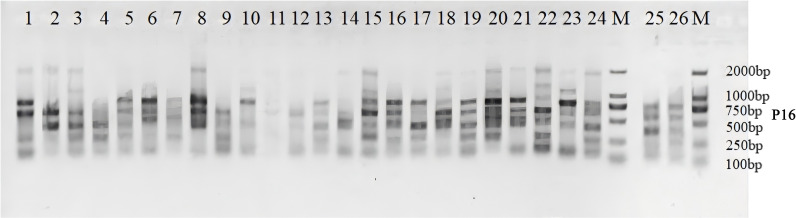
ISSR amplication profile of 26 iris genpotypes generated with primer P16 and separared by 2% agarose gel electrophoresis. M, 100-bp DNA Marker. Lanes 1-26, Iris genotypes listed in **Table 1**.

As illustrated in **Supplementary Table S3**, 9 ISSR random primers amplified a total of 72 DNA bands in Iris accessions, of which 66 were polymorphic. The percentage of polymorphism reached 89.86%, indicating a high level of genetic diversity among the examined cultivars. The total number of DNA loci generated through PCR amplification ranged from 5 to 10. The average number of DNA bands amplified by the nine primers was 8.0, while the mean number of polymorphic bands for ISSR markers was 7.3. The Neis gene diversity (*H*) and Shannons index (*I*) ranged from 0.46 to 0.51 and 0.61 to 0.69, respectively, with mean values of 0.49 and 0.67. These results suggest significant genetic variation within Iris accessions and substantial intra-specific heterogeneity. The polymorphism information content (PIC) values varied among loci, with primer P16 showing the highest value and P9 the lowest, yielding an average PIC of 0.41, indicating moderately effectiveness for germplasm identification and genetic research. **Figure 1** and **Supplementary Figure S1** present the DNA fingerprint profiles obtained after PCR amplification using the selected primers.

### 3.2 SRAP amplification polymorphism analysis

**Figure 2:**
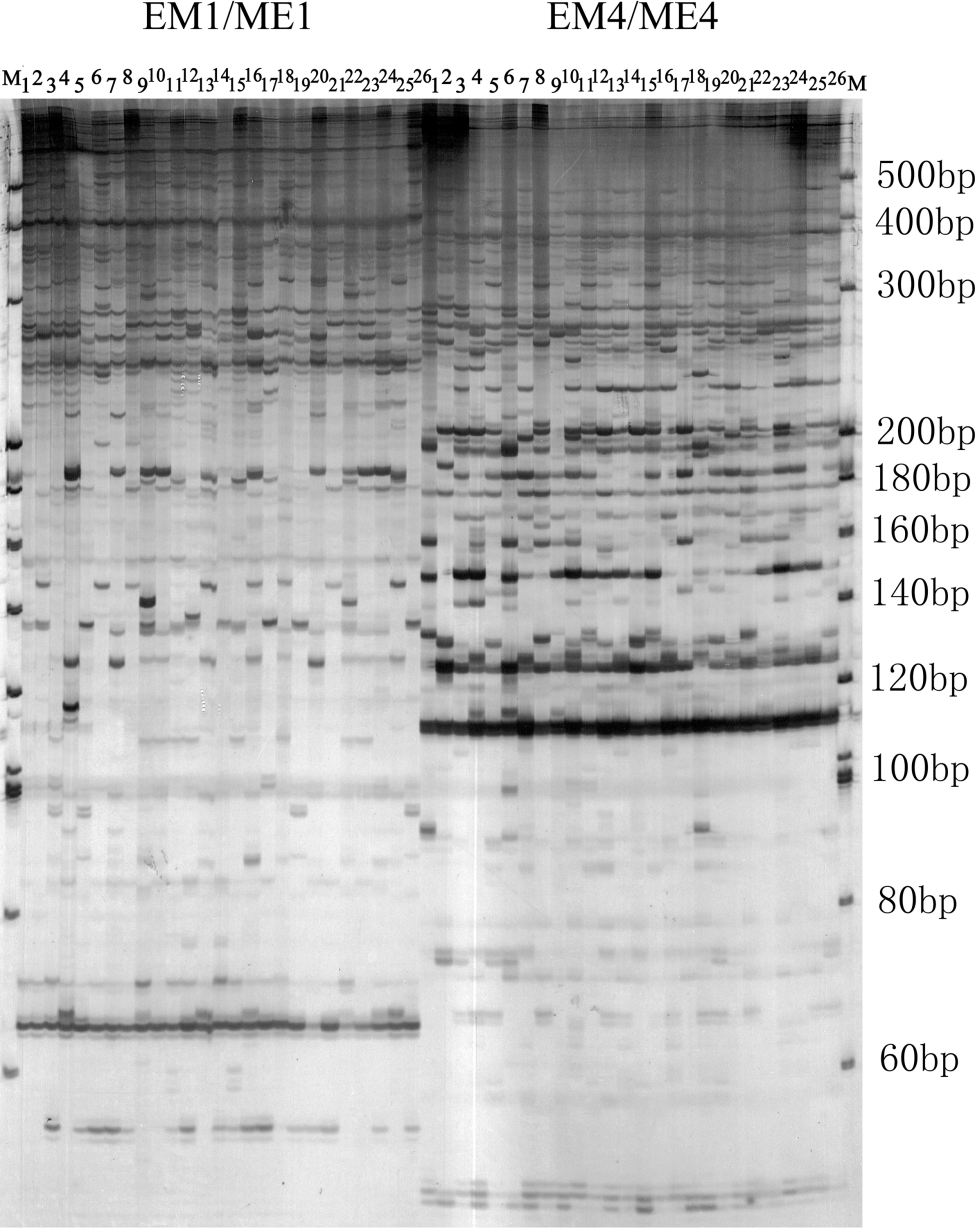
SRAP amplification profiles of 26 iris genotypes generated with prmier pairs EM1ME1 and EM4ME4 and separated by polyacrylamide gel electrophoresis. M: 20-bp DNA Marker. Lanes 1-26; Iris genotypes listed in **Table 1**.

As presented in **Supplementary Table S4**, 12 SRAP primer pairs generated a total of 693 DNA bands in Iris, of which 669 were polymorphic, yielding a polymorphism percentage of 96.54%. This high level of polymorphism indicates substantial intraspecific genetic diversity among Iris accessions. The number of total DNA loci amplified by each primer pair ranged from 40 to 79, with EM7+ME1 producing the highest number of bands and EM1+ME6 the lowest. The average number of DNA bands amplified per primer pair was 57.75, with a mean of 55.75 polymorphic bands per SRAP marker. The Neis gene diversity (*H*) and Shannons index (*I*) ranged from 0.62 to 0.75 and 0.70 to 0.83, respectively, with mean values of 0.68 and 0.76. These results suggest significant inter varietal genetic diversity among Iris. The polymorphism information content (PIC) values varied among loci, with EM7ME1 showing the highest value and EM1ME2 the lowest, yielding an average PIC of 0.63, indicating that the SRAP primers exhibit a high level of polymorphism. **Figure 2** and **Supplementary Figure S2** illustrate representative DNA fingerprinting profiles obtained from PCR amplification using the selected primers.

### 3.3 Genetic diversity analysis

The genetic diversity of 26 *I. germanica* accessions was analyzed using ISSR and SRAP molecular markers (**Supplementary Table S5**, **Supplementary Table S6**). ISSR analysis revealed that the genetic similarity coefficients ranged from 0.465 to 0.838. The highest similarity (0.838) was observed between *I. germanica* Dwarf Dream (No. 22) and Flute Sound (No. 24). In contrast, the lowest similarity (0.465) was found between Blood stone (No. 25) and Antique Red (No. 26).

Similarly, SRAP marker analysis showed genetic similarity coefficients ranging from 0.595 to 0.801. The highest similarity (0.801) was detected between Purple Glow (No. 11) and White Calyx (No. 15), while the lowest similarity (0.595) was observed between Purple Flower (No. 1) and Purple Brown (No. 18).

The experimental results revealed that while Iris plants with the highest coefficient of similarity identified by the ISSR and SRAP marker methods were not identical, they shared similar phenotypic traits, indicating a closer genetic relationship. Conversely, the Iris varieties with the least similarity coefficients from both methods also differed as well. Notably, the Purple Flower (No. 1) and Purple Brown (No. 18) exhibit significant variations in leaf shape, flower color, and flower type under the SRAP method, whereas the Blood stone (No. 25) and Antique Red (No. 26) from the ISSR method represent tall and short types, respectively. Considering their respective appearances, these differences suggested a more distant genetic relationship between the Iris varieties with the lowest similarity coefficients determined by the two marker methods.

### 3.4 Molecular marker cluster analysis

#### 3.4.1 ISSR cluster analysis

**Figure 3:**
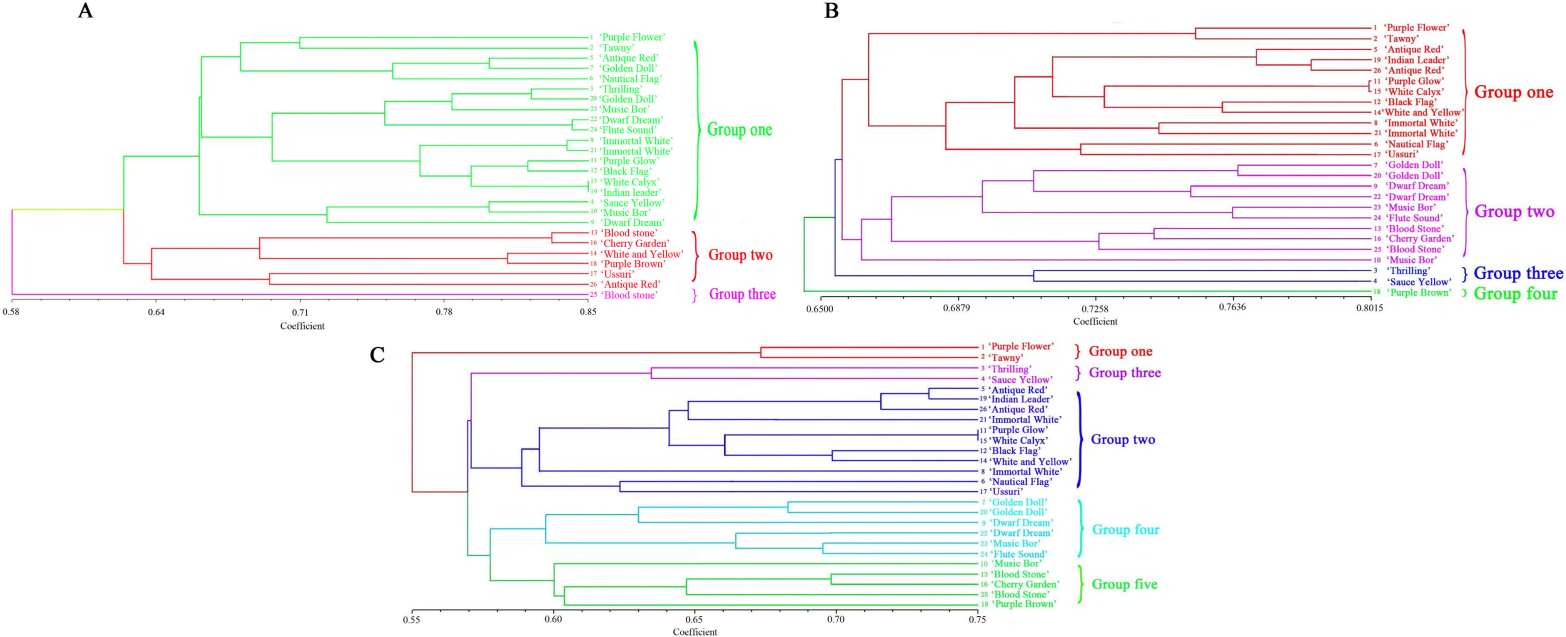
UPGMA dendrogram for 26 *Iris germanica*. **(A)** UPGMA dendrogram based on ISSR amrkers for 26 *Iris germanica.* **(B)** UPGMA dendrogram based on SRAP markers for *Iris germanica*. **(C)** UPGMA dendrogram based on ISSR and SRAP markers for 26 *Iris germanica*.

**Figure 4:**
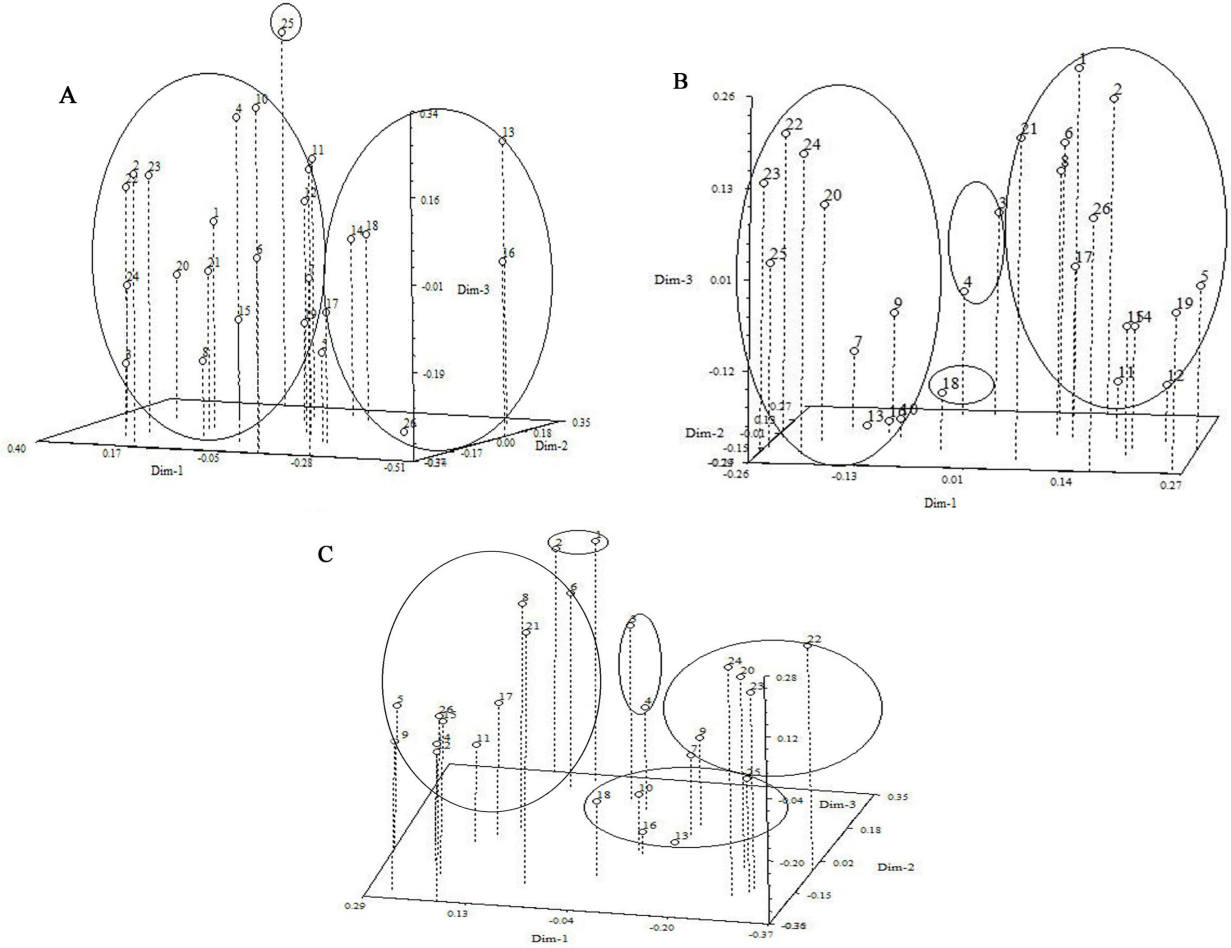
Three- dimensional PCA plot of 26 *Iris germanica* **(A)** Three-dimensional PCA plot derived from ISSR markers of *Iris germnica.* **(B)** Three-dimensional PCA plot derived from ISSR and SRAP markers of 26 *Iris germanica*. **(C)** Three-dimensional PCA plot derived from ISSR and SRAP markers of 26 *Iris germanica*. Numbers 1-26, Iris genotypes listed in **Table 1**.

**Figure 3A** depicted the dendrogram illustrating the molecular clustering of 26 varieties of *I. germanica*. A CCC of 0.65 indicated moderate agreement between the clustering results and the original similarity matrix. The analysis revealed that *I. germanica* could be categorized into 3 main groups at a genetic similarity coefficient of 0.628. Group three comprised a single variety: Blood stone (No. 25). Group two consisted of Antique Red (No. 26), Ussuri (No. 17), Purple Brown (No. 18), White and Yellow (No. 14), Cherry Garden(No. 16) and Blood stone (No. 13). The remaining *I. germanica* varieties fell into the first major group, which were further subdivided into three clusters (0.780). The compact and short-statured *I. germanica* (Sauce Yellow (No. 4), Dwarf Dream (No. 9), and Music Bor (No. 10) were grouped together. The tall and robust Iris varieties (two Immortal White (No. 8 and 21), Black Flag (No. 12), Purple Glow (No. 11), White Calyx (No. 15), Indian Leader (No. 19), Purple Flower (No. 1), Tawny (No. 2), Antique Red (No. 5), and Nautical Flag (No. 6)) were classified into the second and third cluster, respectively (**Figure 4A**).

#### 3.4.2 SRAP cluster analysis

**Figure 3B** displayed the molecular systematic clustering tree of 26 varieties of *I. germanica*. A CCC of 0.70 indicated good agreement between the clustering results and the original similarity matrix. The clustering tree revealed the division of *I. germanica* into 4 major groups at a genetic similarity coefficient of 0.720. The first group comprised 13 tall-stemmed cultivars of *I. germanica* (Purple Flower (No. 1), Tawny (No. 2), Antique Red (No. 5), Indian leader (No. 19), Antique Red (No. 26), Purple Glow (No. 11), White Calyx (No. 15), Black Flag (No. 12), White and Yellow (No. 14), Immortal White (No. 8), Immortal White (No. 21), Nautical Flag (No. 6), Ussuri(No. 17)), while the second group consisted of 10 dwarf species of *I. germanica* (Golden Doll (No. 7), Golden Doll (No. 20), Dwarf Dream (No. 9), Dwarf Dream (No. 22), Music Bor (No. 23), Flute Sound (No. 24), Blood stone (No. 13), Cherry Garden (No. 16), Blood stone(No. 25), Music Bor (No. 10)). The third group included two varieties of *I. germanica* with the same flower color: Thrilling (No. 3) and Sauce Yellow (No. 4). Notably, in the first and second groups, the same varieties of *I. germanica* from different regions, such as Golden Doll (No. 7 and No. 20), Dwarf Dream (No. 9 and No. 22), and Immortal White (No. 21 and No. 8), were first clustered together. The fourth group was composed of a single material of Purple Brown (No. 18) (**Figure 4B**).

#### 3.4.3 SRAP and ISSR comprehensive clustering analysis

By utilizing ISSR and SRAP molecular markers, a clustering analysis was conducted on 26 cultivars of *I. germanica*, leading to the creation of a systematic clustering tree (**Figure 3C**). A CCC value of 0.74 indicated a good clustering result. The analysis revealed that the tested *I. germanica* cultivars fell into 5 major categories when the genetic similarity reached 0.580. The first category consisted of Purple Flower (No. 1) and Yellow Brown (No. 2), both *I. germanica* cultivars originating from Shanxi. The second category included 11 robust *I. germanica* cultivars. The third category comprised two cultivars, Thrilling (No. 3) and Sauce Yellow (No. 4), with identical flower colors. The fourth category was characterized by 6 dwarf *I. germanica* cultivars. Lastly, the fifth category encompassed 5 *I. germanica* materials, among which were three short cultivars with the same flower colors (No. 13, No. 16, No. 25) (**Figure 4C**).

The results of three clustering analyses demonstrated that utilizing ISSR and SRAP clustering methods independently could effectively differentiate the 26 experimental samples of *I. germanica* based on their morphological characteristics. Particularly, SRAP clustering could group the same *I. germanica* varieties from different regions together, with slight variations in genetic distances. However, when dealing with different *I. germanica* types from the same region, only by combining both molecular markers could be distinctly separated the two *I. germanica* materials from Shanxi Province. Notably, all three clustering methods grouped *I. germanica* Purple Glow, White Calyx, and Black Flag respectively in close proximity, indicating minimal genetic differentiation.

The AMOVA results of *I. germanica* cultivars (**Supplementary Table S7**) indicated a certain degree of genetic differentiation among different populations. Of the total genetic variation, only 18.32% was attributed to differences among populations, while 81.68% of the variation occurred within populations. The significantly higher proportion of genetic variation within populations suggested that cultivar variation was the primary source of genetic diversity in *I. germanica*.

## 4 Discussion

A total of 72 loci were detected by screening 9 ISSR primers, of which 66 exhibited polymorphic bands. This result aligns with the findings of Zhang on *I. germanica* using ISSR analysis (Zhang et al., 2017), indicating the efficacy of ISSR markers in assessing the genetic diversity of iris materials. The high level of polymorphism of ISSR primers observed in this study (89.86%) was comparable to reports in other *Iris* species, such as *I. lactea* (77.29%; Mao et al., 2013) and *I. germanica* (89.7%; Zhang et al., 2017). These findings collectively demonstrated that the genus Iris exhibits substantial genetic variation in inter-SSR regions, which was advantageous for cultivar identification and kinship analysis using ISSR molecular markers. However, as ISSR markers primarily reflected polymorphism in non-coding genomic regions, their utility in assessing functional gene-associated diversity might be limited.

Additionally, 12 pairs of SRAP primers identified a remarkable 693 loci, with 669 showing polymorphic bands, surpassing the polymorphism observed with ISSR markers. This discrepancy likely stemmed from fundamental differences in their detection mechanisms. In contrast to ISSR, the SRAP marker employed a pair of primers (the forward primer targets the GC-rich exon regions, while the reverse primer targets the AT-rich intron/promoter regions), covered a wider proportion of the functional genome, and generated a higher number of polymorphic bands per reaction. Moreover, the polymorphisms detected by SRAP markers showed higher probability of direct association with functional genomic variations, particularly in coding and regulatory sequences. The high polymorphism rate of SRAP primer pairs (96.54%) observed in this study indicated that German iris cultivars harbored rich genetic diversity within gene-coding and regulatory regions, which correlated with their prolonged artificial domestication, extensive hybrid breeding history, and global dissemination as ornamental plants. Compared with our findings, Xu et al. (2015) and Tong et al. (2019) similarly employed SRAP markers to analyze iris germplasm, reporting a polymorphism rate of 100%, possibly due to the presence of wild iris accessions in their collections.

Generally, a marker is considered highly polymorphic when PIC > 0.50, while PIC < 0.25 indicates low polymorphism (Sharma et al., 2021). Among the tested materials, SRAP primers (PIC = 0.63) were classified as high-polymorphism markers, exhibiting greater polymorphism compared to ISSR primers (PIC = 0.41), which indicated that SRAP primers contained more polymorphic information. Wei et al. (2023) reported an average PIC value of 0.67 in *Aegilops tauschii* using ISSR markers, while Tang et al. (2015) observed exceptionally high polymorphism (PIC = 0.91-0.95) among 17 SRAP primer pairs in *Cymbidium*. Although compared with other species mentioned above, the PIC values of both markers in *I. germanica* were relatively lower, they aligned with reported PIC values for flax cultivars (PIC = 0.61; Yi et al., 2018) and wheat cultivars (PIC = 0.28; Abouseada et al., 2023). This may be related to the fact that all the *I. germanica* germplasm materials used in this study were cultivated varieties. In the future, the scope of collecting varieties of *I. germanica* can be expanded to enrich the genetic diversity of the resources. In the meantime, The Neis gene diversity (*H* = 0.68) and Shannons index (*I* = 0.76) obtained from SRAP were higher than those from ISSR (*H* = 0.49 and *I* = 0.67), as well as greater than those of 8 Iris germplasms from Liaoning (*H* = 0.24 and *I* = 0.16; Di et al., 2022) and 15 Iris germplasms introduced from northern China (*H* = 0.40 and *I* = 0.58; Xu et al., 2015). This indicated that SRAP markers had higher efficiency and that the tested germplasm possessed richer genetic diversity.

ISSR and SRAP markers, being dominant and codominant respectively, produce different sequences during amplification. Utilizing both marker types allows for a comprehensive assessment of genetic diversity in the test materials from diverse perspectives (Godwin et al., 1997; Li and Quiros, 2001). The simultaneous use of both markers can increase the number of polymorphic loci, particularly enhancing the detection of polymorphic loci compared to using ISSR markers alone. The integration of SRAP markers ensures a more thorough and nuanced representation of DNA information, leading to more robust and scientifically valid outcomes. However, this combined marker approach requires consideration of inherent limitations. As both ISSR and SRAP are dominant markers, their inability to discriminate heterozygotes may lead to underestimation of genetic diversity. Variations in primer amplification efficiency may introduce systematic biases in population genetic diversity assessments. Therefore, to account for these technical constraints, integrating phenotypic data for cross-validation is recommended to enhance the reliability and comprehensiveness of research conclusions.

The clustering analysis based on ISSR markers revealed that 26 samples of *I. germanica* were primarily categorized by shared morphological features rather than flower color or shape. The phenotypic traits of these *I. germanica* cultivars were shown in **Supplementary Table S8** and **Supplementary Table S9**. This indicated the suitability of utilizing ISSR markers for assessing genetic diversity in *I. germanica*. Cultivars such as Black Flag, Immortal White, and Bloodstone, Cherry Garden displayed close genetic relationships with relatively small genetic distances. The clustering results from SRAP markers indicated two iris varieties (Purple Flower and Yellow Brown) from Shanxi province were grouped into a single cluster, suggesting that there was a phenomenon of resource exchange among iris varieties in Shanxi region. Furthermore, on the basis of grouping iris cultivars with similar phenotypes into one category, the same varieties from different region were first clustered together, such as the Immortality White introduced from Henan and Hebei province, the Golden Doll and Dwarf Dream introduced from Liaoning and Hebei province. This highlighted the precision of SRAP markers in assessing genetic diversity. The integrated clustering results of ISSR and SRAP not only grouped morphologically similar iris cultivars together but also separated two iris materials from Shanxi region. Tong (Tong et al., 2015) similarly utilized molecular markers to group the same dwarf iris cultivars: Golden Doll, Dwarf Dream, Flute Sound and Music Bor into one category, indicating that these varieties were closely related.

Particularly, *I. germanica* Bloodstone and Cherry Garden, sharing identical flower colors, flower characteristics and plant types, were consistently grouped together across all three molecular markers. The earlier breeding history of Bloodstone in comparison to Cherry Garden suggested the latter may be a hybrid descendant of the former. These findings further supported the correlation between phenotypic similarity and genetic background. This result demonstrated the reliability of molecular markers for distinguishing closely related cultivars, particularly those with nearly identical phenotypes. Moreover, the genetic clustering suggests these two cultivars may share common parental origins, providing valuable references for breeding practices. Furthermore, the combined clustering analysis using both molecular markers effectively distinguished between dwarf and tall iris cultivars, which was consistent with the clustering results based on phenotypic traits (**Supplementary Figure S3**).

Moreover, this research indicated that two *I. germanica* cultivars native to Shanxi showed distant genetic relationships compared to cultivars from other regions. Due to the extensive genetic diversity within the iris genus, especially among *I. germanica* cultivars, each cultivar possessed a significant number of parentages (Huang et al., 2003). Furthermore, limited gene exchange among plants in different regions, influenced by geographical barriers, resulted in genetic divergence (Han et al., 2015). Therefore, it was postulated that these cultivars might have been developed through hybridization and selective breeding among iris plants, particularly *I. germanica*, cultivated in Shanxi over an extended period.

As a horticultural cultivar, *I. germanica* possesses a complex genetic background. The high polymorphism levels of the primers observed in this study, particularly with SRAP markers (96.54%), demonstrated substantial genetic variation within its germplasm resources. Strategic selection of genetically distant and complementary parental combinations (Nautical Flag (No. 6) and Dwarf Dream(No. 22); Purple Flower (No. 1) and Purple Brown (No. 18)) could effectively enhance both genetic diversity and trait performance in hybrid progeny. Moreover, the abundant polymorphic loci provided ample marker resources for marker-assisted selection (MAS), while the identification of core primers (EM7ME1) offered efficient analytical tools for subsequent genetic analyses. Crucially, SRAP markers preferentially detected polymorphisms in functional genomic regions, facilitating efficient identification of loci associated with key ornamental traits such as flower coloration, morphology, and disease resistance for targeted genetic improvement. Despite the existing diversity among cultivars, the high polymorphism levels revealed in this study also underscored the need to establish specialized germplasm banks to prioritize the collection and conservation of germplasm carrying rare alleles, thereby preventing the loss of valuable genetic variation due to selection pressures during breeding processes. Furthermore, continuous monitoring of gene flow between cultivated populations and wild relatives was imperative to safeguard against genetic erosion (Cheng and Bao, 2001).

## 5 Conclusions

In conclusion, the high percentage of polymorphic bands obtained through ISSR (89.86%) and SRAP (96.54%) marker analyses indicated substantial intra-specific genetic diversity of 26 *I. germanica* accessions. The genetic similarity coefficients obtained from both marker systems showed a definite genetic connection among the different *I. germanica* cultivars, and it was observed that cultivars with a closer genetic relationship tended to display analogous phenotypic traits. The molecular marker cluster analysis further supported the genetic diversity findings, with *I. germanica* cultivars being grouped into distinct clusters based on their genetic similarities, and the clustering results were essentially consistent with their morphological characteristics. Additionally, the combined use of ISSR and SRAP markers proved effective in isolating two *I. germanica* species from Shanxi province. This study offers valuable insights into the genetic diversity and relationships within iris species, providing a foundation for further research and practical applications in the field of ornamental plant breeding and conservation.

## Data Availability

The datasets presented in this study can be found in online repositories. The names of the repository/repositories and accession number(s) can be found in the article/**Supplementary Material**.
