# Supplementary material for: Genetic diversity analysis of Iris germanica cultivars based on ISSR and SRAP molecular markers
Source: Front Plant Sci. 2025 Sep 10;16:1629234. doi: 10.3389/fpls.2025.1629234 (PMC12457380; doi:10.3389/fpls.2025.1629234)
Supplement: Supplementary file 1 [file DataSheet1.docx]

Supplementary Material

# Supplementary Figures

*
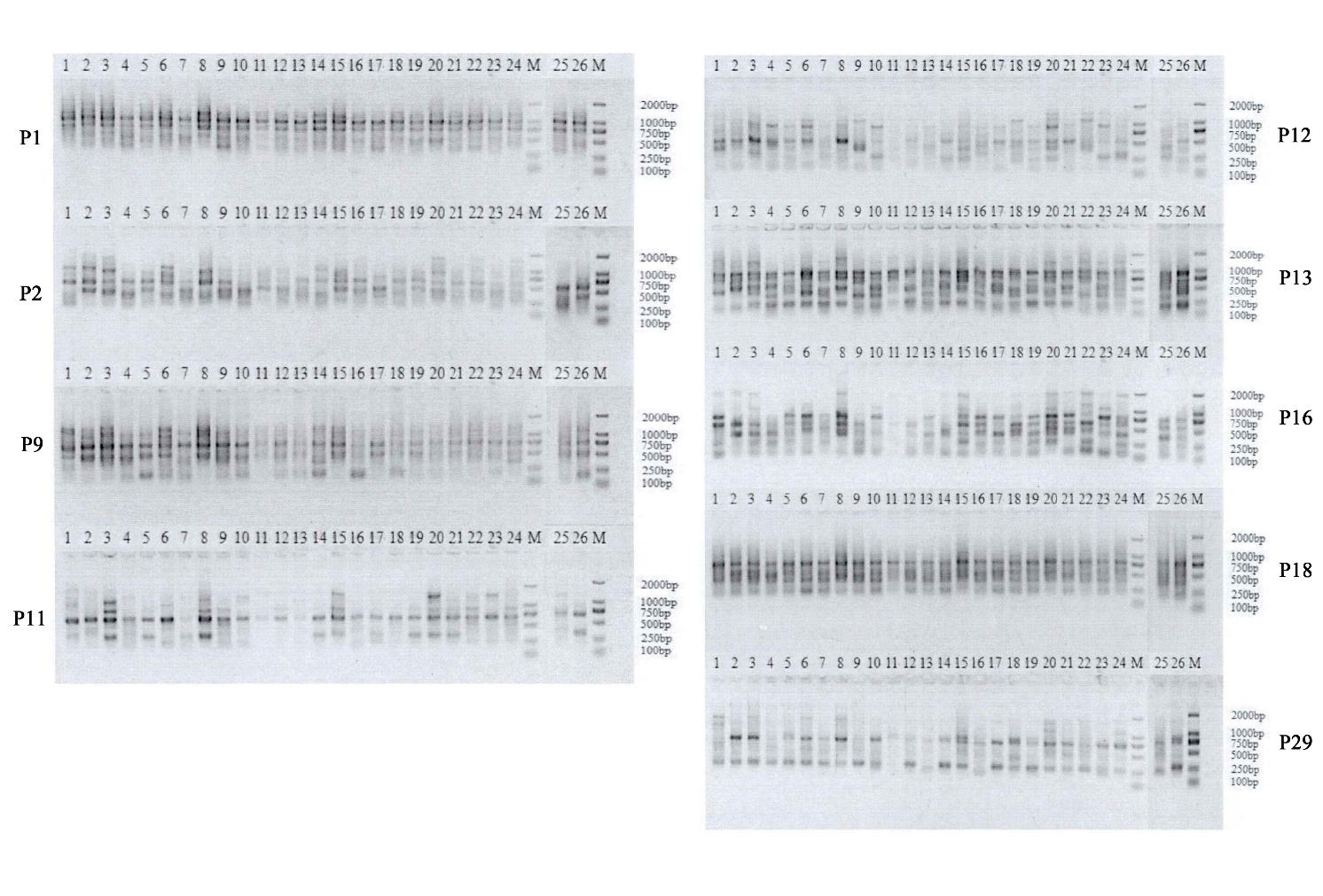
*

Figure S1 Amplification results of ISSR Primer.


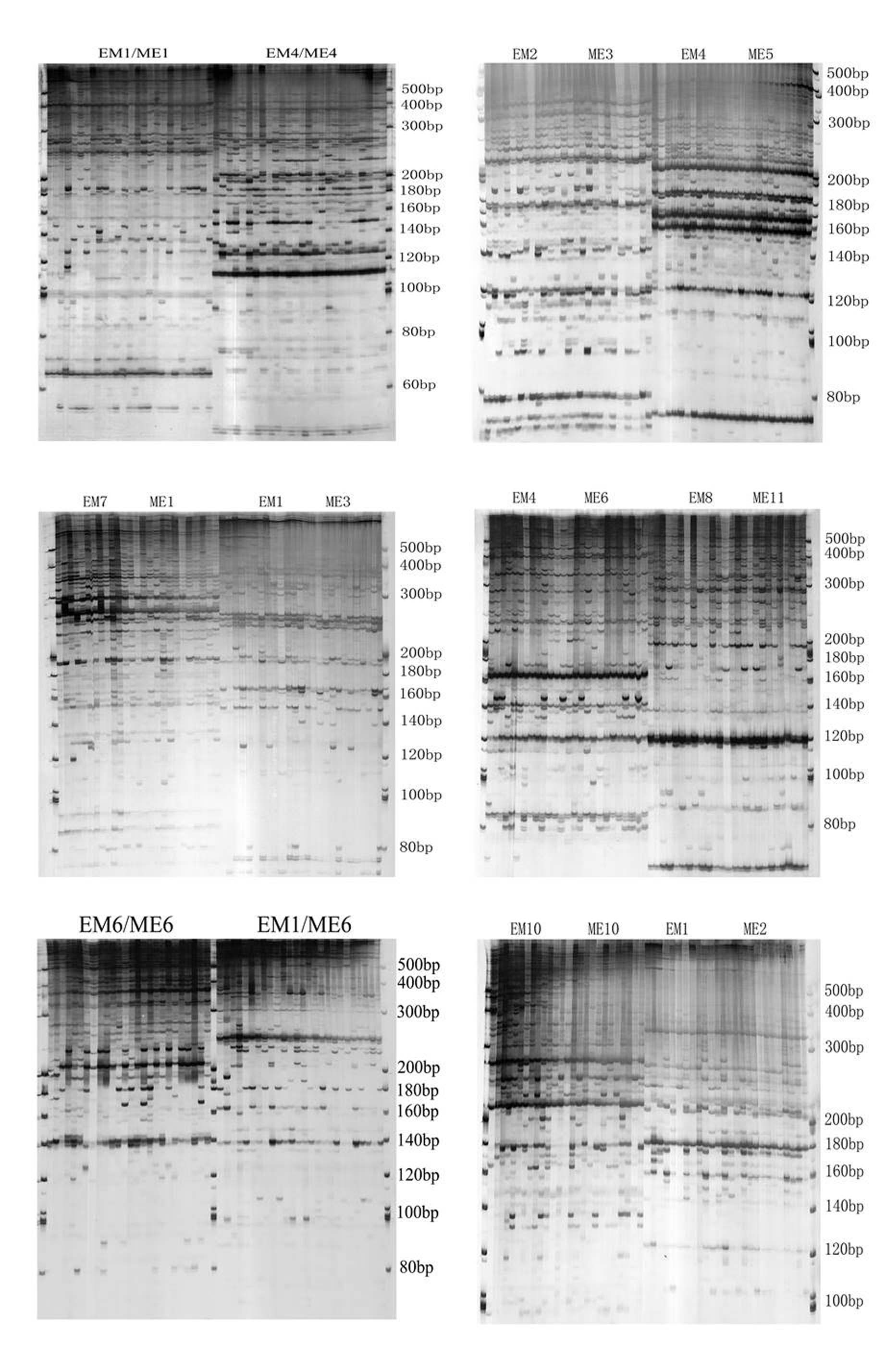


Figure S2 Amplification results of SRAP Primer.


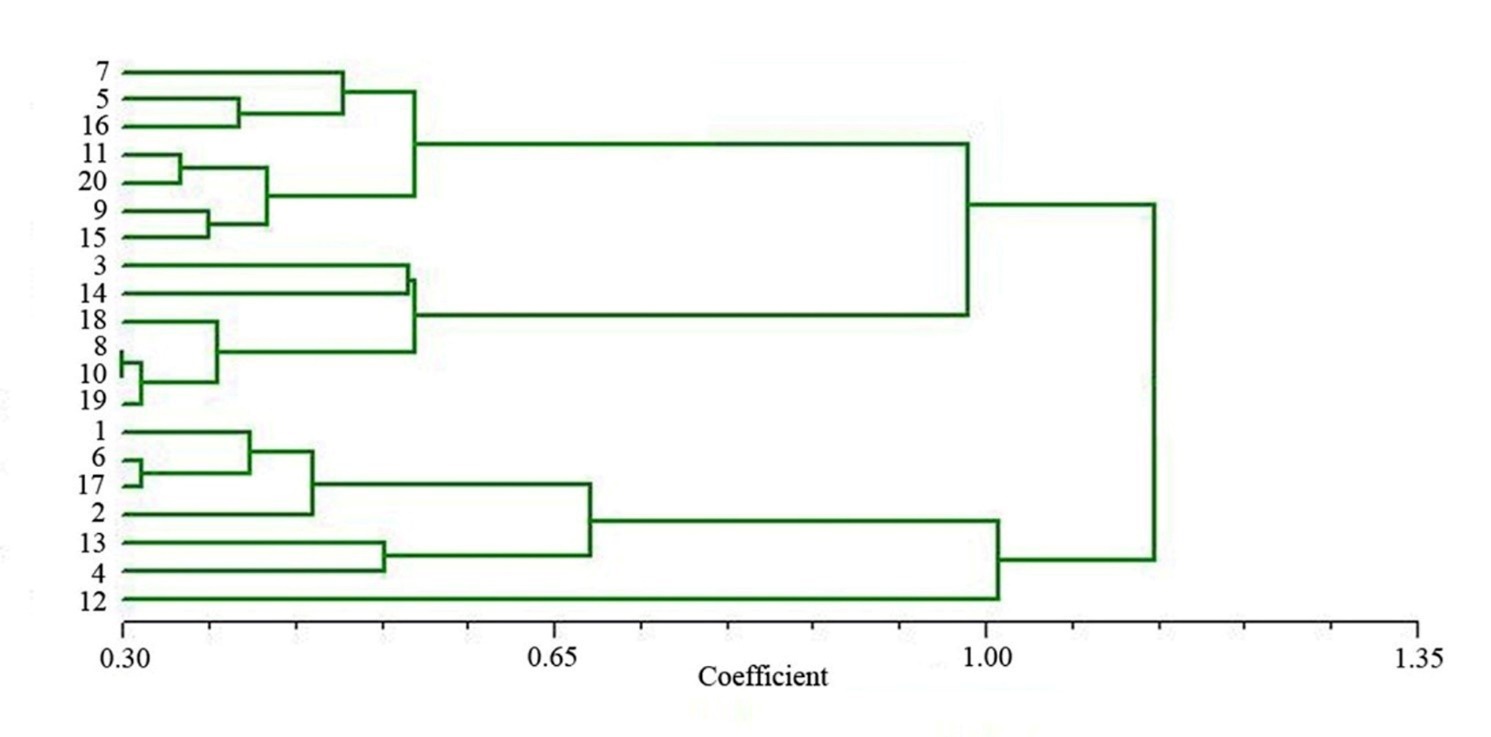


Figure S3 UPGMA Cluster based on average taxonomic distance (Dist) morphological traits in *Iris germanica*. 1: *Iris germanica* ‘Purple Flower’. 2: ‘Tawny’. 3: ‘Thrilling’. 4: ‘Antique Red’. 5: ‘Golden Doll’. 6: ‘Nautical Flag’. 7: ‘Dwarf Dream’. 8: ‘Purple Glow’. 9: ‘Bloodstone’. 10: ‘White Calyx’. 11: ‘Sauce Yellow’. 12: ‘Immortality’. 13: ‘Black Flag’. 14: ‘White and Yellow’. 15: ‘Cherry Garden’. 16: ‘Music Bor’. 17: ‘Ussuri’. 18: ‘Purple Brown’. 19: ‘Indian leader’. 20: ‘Flute Sound’.

# Supplementary Tables

Table S1 The primers of ISSR

| Code | Sequences | Code | Sequences |
| --- | --- | --- | --- |
| P1 | (GGGGT)_3_ | P21 | (CA)_6_GT |
| P2 | (GA)_8_T | P22 | (GT)_8_YC |
| P3 | (TC)_8_G | P23 | (CTC)_6_ |
| P4 | (CT)_8_RC | P24 | (GT)_8_C |
| P5 | (CT)_8_RG | P25 | (GT)_8_GG |
| P6 | (GT)_8_YG | P26 | G(AC)_8_ |
| P7 | (TC)_8_RT | P27 | (CT)_8_A |
| P8 | (TC)_8_RG | P28 | (CT)_8_T |
| P9 | (TG)_8_RC | P29 | (GGAGA)_3_ |
| P10 | (TG)_8_RA | P30 | (AG)_8_YG |
| P11 | (GACA)_4_ | P31 | (AG)_8_A |
| P12 | (GA)_8_YT | P32 | (GA)_8_RG |
| P13 | (AG)_8_YC | P33 | (GT)_8_YA |
| P14 | (AG)_8_YT | P34 | (GT)_8_RG |
| P15 | (AG)_8_RA | P35 | (GT)_8_YT |
| P16 | (GA)_8_YC | P36 | (AC)_8_YG |
| P17 | (GA)_8_YA | P37 | (AC)_8_YA |
| P18 | (AC)_8_RG | P38 | (GTG)_5_ |
| P19 | (GTGC)_4_ | P39 | (AC)_8_RA |
| P20 | (CCT)_5_ | P40 | (AC)_8_RT |

Y：C/T；R：A/G

Table S2 The primers of SRAP

| Code | Forward primers sequences | Code | Reverse primers sequences |
| --- | --- | --- | --- |
| ME1 | 5'-TGAGTCCAAACCGGATC-3’ | EM1 | 5’-GACTGCGTACGAATTAAT-3’ |
| ME2 | 5’-TGAGTCCAAACCGGAGC-3’ | EM2 | 5’-GACTGCGTACGAATTTGC-3’ |
| ME3 | 5’-TGAGTCCAAACCGGAAT-3’ | EM3 | 5’-GACTGCGTACGAATTGAC-3’ |
| ME4 | 5’-TGAGTCCAAACCGGATA-3’ | EM4 | 5’-GACTGCGTACGAATTGCG-3’ |
| ME5 | 5’-TGAGTCCAAACCGGTCC-3’ | EM5 | 5’-GACTGCGTACGAATTAAC-3’ |
| ME6 | 5’-TGAGCTCTTTCCGGTAA-3’ | EM6 | 5’-GACTGCGTACGAATTCAA-3’ |
| ME7 | 5’-TGAGCTCTTTCCGGTTG-3’ | EM7 | 5’-GACTGCGTACGAATTATG-3’ |
| ME8 | 5’-TGAGTCCAAACCGGTGC-3’ | EM8 | 5’-GACTGCGTACGAATTAGC-3’ |
| ME9 | 5’-ACAGTCGAAACCGGTCA-3’ | EM9 | 5’-GACTGCGTACGAATTCAG-3’ |
| ME10 | 5’-ACAGTGGAAACGCGTAC-3’ | EM10 | 5’-GACTGCGTACGAATTTAG-3’ |
| ME11 | 5’-TGAGTCCAAACCGGAAG-3’ | EM11 | 5’-GACTGCGTACGAATTTCG-3’ |
|  |  | EM12 | 5’-GACTGCGTACGAATTCAT-3’ |
|  |  | EM13 | 5’-GACTGCGTACGAATTACT-3’ |
|  |  | EM14 | 5’-GACTGCGTACGAATTCCA-3’ |

Table S3 ISSR primers and their diversity statistics

| Primer | Sample Size | Shannon’s Information Index(*I*) | Nei’s gene diversity(*H*) | Polymorphism Information Content (PIC) | Number of  fragments scored | Polymorphic  fragments | Ratio of polymorphism |
| --- | --- | --- | --- | --- | --- | --- | --- |
| P1 | 26 | 0.69 | 0.51 | 0.42 | 6 | 4 | 66.67% |
| P2 | 26 | 0.67 | 0.48 | 0.40 | 8 | 8 | 100.00% |
| P9 | 26 | 0.61 | 0.46 | 0.30 | 8 | 7 | 87.50% |
| P11 | 26 | 0.69 | 0.51 | 0.40 | 9 | 8 | 88.89% |
| P12 | 26 | 0.69 | 0.50 | 0.47 | 10 | 10 | 100.00% |
| P13 | 26 | 0.67 | 0.48 | 0.44 | 10 | 10 | 100.00% |
| P16 | 26 | 0.69 | 0.50 | 0.50 | 9 | 9 | 100.00% |
| P18 | 26 | 0.69 | 0.51 | 0.40 | 5 | 4 | 80.00% |
| P29 | 26 | 0.61 | 0.46 | 0.33 | 7 | 6 | 85.71% |
| sum total | 26 |  |  |  | 72 | 66 |  |
| mean value |  | 0.67 | 0.49 | 0.41 | 8.0 | 7.3 | 89.86% |

| Primer | Sample Size | Shannon’s Information Index(*I*) | Nei’s gene diversity(*H*) | Polymorphism Information Content (PIC) | Number of  fragments scored | Polymorphic  fragments | Ratio of polymorphism |
| --- | --- | --- | --- | --- | --- | --- | --- |
| EM1ME1 | 26 | 0.75 | 0.67 | 0.65 | 66 | 63 | 95.45% |
| EM1ME2 | 26 | 0.70 | 0.62 | 0.48 | 55 | 54 | 98.18% |
| EM1ME3 | 26 | 0.78 | 0.70 | 0.65 | 45 | 43 | 95.56% |
| EM1ME6 | 26 | 0.72 | 0.64 | 0.60 | 40 | 36 | 90.00% |
| EM2ME3 | 26 | 0.76 | 0.68 | 0.65 | 57 | 56 | 98.25% |
| EM4ME4 | 26 | 0.80 | 0.72 | 0.67 | 71 | 67 | 94.37% |
| EM4ME5 | 26 | 0.74 | 0.66 | 0.62 | 43 | 37 | 86.05% |
| EM4ME6 | 26 | 0.75 | 0.67 | 0.63 | 67 | 66 | 98.51% |
| EM6ME6 | 26 | 0.78 | 0.70 | 0.64 | 46 | 45 | 97.83% |
| EM7ME1 | 26 | 0.83 | 0.75 | 0.70 | 79 | 79 | 100.00% |
| EM8ME11 | 26 | 0.76 | 0.68 | 0.63 | 61 | 61 | 100.00% |
| EM10ME10 | 26 | 0.73 | 0.65 | 0.61 | 63 | 62 | 98.41% |
| sum total | 26 |  |  |  | 693 | 669 |  |
| mean value |  | 0.76 | 0.68 | 0.63 | 57.75 | 55.75 | 96.54% |

Table S4 SRAP primers and their diversity statistics

Table S5 Genetic similarity coefficient matrix of 26 iris resources based on ISSR molecular markers

|  | 1 | 2 | 3 | 4 | 5 | 6 | 7 | 8 | 9 | 10 | 11 | 12 | 13 | 14 | 15 | 16 | 17 | 18 | 19 | 20 | 21 | 22 | 23 | 24 | 25 | 26 |
| --- | --- | --- | --- | --- | --- | --- | --- | --- | --- | --- | --- | --- | --- | --- | --- | --- | --- | --- | --- | --- | --- | --- | --- | --- | --- | --- |
| 1 | 1.000 |  |  |  |  |  |  |  |  |  |  |  |  |  |  |  |  |  |  |  |  |  |  |  |  |  |
| 2 | 0.711 | 1.000 |  |  |  |  |  |  |  |  |  |  |  |  |  |  |  |  |  |  |  |  |  |  |  |  |
| 3 | 0.721 | 0.733 | 1.000 |  |  |  |  |  |  |  |  |  |  |  |  |  |  |  |  |  |  |  |  |  |  |  |
| 4 | 0.641 | 0.692 | 0.629 | 1.000 |  |  |  |  |  |  |  |  |  |  |  |  |  |  |  |  |  |  |  |  |  |  |
| 5 | 0.642 | 0.654 | 0.771 | 0.693 | 1.000 |  |  |  |  |  |  |  |  |  |  |  |  |  |  |  |  |  |  |  |  |  |
| 6 | 0.724 | 0.736 | 0.711 | 0.627 | 0.740 | 1.000 |  |  |  |  |  |  |  |  |  |  |  |  |  |  |  |  |  |  |  |  |
| 7 | 0.666 | 0.679 | 0.654 | 0.680 | 0.800 | 0.769 | 1.000 |  |  |  |  |  |  |  |  |  |  |  |  |  |  |  |  |  |  |  |
| 8 | 0.711 | 0.724 | 0.800 | 0.615 | 0.763 | 0.701 | 0.679 | 1.000 |  |  |  |  |  |  |  |  |  |  |  |  |  |  |  |  |  |  |
| 9 | 0.642 | 0.581 | 0.596 | 0.693 | 0.692 | 0.629 | 0.680 | 0.690 | 1.000 |  |  |  |  |  |  |  |  |  |  |  |  |  |  |  |  |  |
| 10 | 0.561 | 0.714 | 0.620 | 0.800 | 0.679 | 0.690 | 0.745 | 0.678 | 0.754 | 1.000 |  |  |  |  |  |  |  |  |  |  |  |  |  |  |  |  |
| 11 | 0.653 | 0.705 | 0.641 | 0.622 | 0.708 | 0.600 | 0.608 | 0.745 | 0.666 | 0.693 | 1.000 |  |  |  |  |  |  |  |  |  |  |  |  |  |  |  |
| 12 | 0.615 | 0.666 | 0.679 | 0.755 | 0.791 | 0.640 | 0.652 | 0.705 | 0.750 | 0.775 | 0.818 | 1.000 |  |  |  |  |  |  |  |  |  |  |  |  |  |  |
| 13 | 0.627 | 0.520 | 0.500 | 0.590 | 0.638 | 0.571 | 0.622 | 0.520 | 0.680 | 0.625 | 0.697 | 0.697 | 1.000 |  |  |  |  |  |  |  |  |  |  |  |  |  |
| 14 | 0.716 | 0.576 | 0.592 | 0.608 | 0.653 | 0.627 | 0.638 | 0.653 | 0.612 | 0.600 | 0.711 | 0.666 | 0.681 | 1.000 |  |  |  |  |  |  |  |  |  |  |  |  |
| 15 | 0.701 | 0.678 | 0.724 | 0.640 | 0.716 | 0.545 | 0.588 | 0.785 | 0.679 | 0.629 | 0.816 | 0.775 | 0.583 | 0.680 | 1.000 |  |  |  |  |  |  |  |  |  |  |  |
| 16 | 0.520 | 0.530 | 0.549 | 0.558 | 0.739 | 0.666 | 0.727 | 0.571 | 0.652 | 0.638 | 0.666 | 0.666 | 0.829 | 0.651 | 0.595 | 1.000 |  |  |  |  |  |  |  |  |  |  |
| 17 | 0.600 | 0.530 | 0.666 | 0.651 | 0.695 | 0.625 | 0.636 | 0.653 | 0.608 | 0.680 | 0.666 | 0.714 | 0.585 | 0.511 | 0.723 | 0.650 | 1.000 |  |  |  |  |  |  |  |  |  |
| 18 | 0.703 | 0.566 | 0.654 | 0.638 | 0.760 | 0.692 | 0.666 | 0.679 | 0.640 | 0.627 | 0.739 | 0.695 | 0.711 | 0.808 | 0.666 | 0.727 | 0.636 | 1.000 |  |  |  |  |  |  |  |  |
| 19 | 0.618 | 0.629 | 0.714 | 0.666 | 0.823 | 0.566 | 0.653 | 0.703 | 0.666 | 0.653 | 0.765 | 0.808 | 0.652 | 0.750 | 0.846 | 0.666 | 0.622 | 0.734 | 1.000 |  |  |  |  |  |  |  |
| 20 | 0.733 | 0.677 | 0.819 | 0.603 | 0.678 | 0.655 | 0.592 | 0.677 | 0.607 | 0.596 | 0.692 | 0.692 | 0.588 | 0.716 | 0.736 | 0.560 | 0.560 | 0.740 | 0.763 | 1.000 |  |  |  |  |  |  |
| 21 | 0.750 | 0.727 | 0.807 | 0.693 | 0.730 | 0.666 | 0.640 | 0.836 | 0.692 | 0.679 | 0.791 | 0.833 | 0.638 | 0.653 | 0.792 | 0.565 | 0.695 | 0.680 | 0.784 | 0.750 | 1.000 |  |  |  |  |  |
| 22 | 0.666 | 0.644 | 0.721 | 0.603 | 0.571 | 0.482 | 0.518 | 0.610 | 0.714 | 0.596 | 0.615 | 0.615 | 0.509 | 0.603 | 0.701 | 0.480 | 0.480 | 0.629 | 0.654 | 0.766 | 0.678 | 1.000 |  |  |  |  |
| 23 | 0.631 | 0.714 | 0.758 | 0.760 | 0.716 | 0.654 | 0.666 | 0.678 | 0.641 | 0.777 | 0.693 | 0.734 | 0.500 | 0.680 | 0.703 | 0.553 | 0.638 | 0.705 | 0.692 | 0.807 | 0.679 | 0.736 | 1.000 |  |  |  |
| 24 | 0.645 | 0.655 | 0.761 | 0.654 | 0.655 | 0.600 | 0.607 | 0.721 | 0.655 | 0.644 | 0.666 | 0.629 | 0.452 | 0.690 | 0.779 | 0.500 | 0.576 | 0.678 | 0.736 | 0.741 | 0.689 | 0.838 | 0.779 | 1.000 |  |  |
| 25 | 0.640 | 0.571 | 0.549 | 0.604 | 0.565 | 0.500 | 0.409 | 0.571 | 0.565 | 0.553 | 0.761 | 0.571 | 0.585 | 0.604 | 0.638 | 0.500 | 0.500 | 0.681 | 0.577 | 0.600 | 0.608 | 0.600 | 0.638 | 0.576 | 1.000 |  |
| 26 | 0.566 | 0.500 | 0.629 | 0.608 | 0.775 | 0.588 | 0.638 | 0.730 | 0.653 | 0.560 | 0.577 | 0.622 | 0.636 | 0.695 | 0.720 | 0.744 | 0.697 | 0.680 | 0.708 | 0.603 | 0.612 | 0.528 | 0.560 | 0.618 | 0.465 | 1.000 |

Table S6 Genetic similarity coefficient matrix of 26 iris resources based on SRAP molecular markers

|  | 1 | 2 | 3 | 4 | 5 | 6 | 7 | 8 | 9 | 10 | 11 | 12 | 13 | 14 | 15 | 16 | 17 | 18 | 19 | 20 | 21 | 22 | 23 | 24 | 25 | 26 |
| --- | --- | --- | --- | --- | --- | --- | --- | --- | --- | --- | --- | --- | --- | --- | --- | --- | --- | --- | --- | --- | --- | --- | --- | --- | --- | --- |
| 1 | 1.000 |  |  |  |  |  |  |  |  |  |  |  |  |  |  |  |  |  |  |  |  |  |  |  |  |  |
| 2 | 0.753 | 1.000 |  |  |  |  |  |  |  |  |  |  |  |  |  |  |  |  |  |  |  |  |  |  |  |  |
| 3 | 0.647 | 0.688 | 1.000 |  |  |  |  |  |  |  |  |  |  |  |  |  |  |  |  |  |  |  |  |  |  |  |
| 4 | 0.623 | 0.651 | 0.709 | 1.000 |  |  |  |  |  |  |  |  |  |  |  |  |  |  |  |  |  |  |  |  |  |  |
| 5 | 0.644 | 0.680 | 0.674 | 0.683 | 1.000 |  |  |  |  |  |  |  |  |  |  |  |  |  |  |  |  |  |  |  |  |  |
| 6 | 0.674 | 0.693 | 0.678 | 0.649 | 0.684 | 1.000 |  |  |  |  |  |  |  |  |  |  |  |  |  |  |  |  |  |  |  |  |
| 7 | 0.603 | 0.602 | 0.645 | 0.639 | 0.651 | 0.612 | 1.000 |  |  |  |  |  |  |  |  |  |  |  |  |  |  |  |  |  |  |  |
| 8 | 0.667 | 0.671 | 0.674 | 0.651 | 0.688 | 0.710 | 0.636 | 1.000 |  |  |  |  |  |  |  |  |  |  |  |  |  |  |  |  |  |  |
| 9 | 0.660 | 0.649 | 0.672 | 0.681 | 0.661 | 0.671 | 0.719 | 0.678 | 1.000 |  |  |  |  |  |  |  |  |  |  |  |  |  |  |  |  |  |
| 10 | 0.602 | 0.626 | 0.606 | 0.635 | 0.638 | 0.628 | 0.658 | 0.623 | 0.677 | 1.000 |  |  |  |  |  |  |  |  |  |  |  |  |  |  |  |  |
| 11 | 0.654 | 0.675 | 0.658 | 0.667 | 0.713 | 0.662 | 0.655 | 0.701 | 0.711 | 0.694 | 1.000 |  |  |  |  |  |  |  |  |  |  |  |  |  |  |  |
| 12 | 0.652 | 0.668 | 0.648 | 0.645 | 0.711 | 0.655 | 0.648 | 0.662 | 0.684 | 0.667 | 0.745 | 1.000 |  |  |  |  |  |  |  |  |  |  |  |  |  |  |
| 13 | 0.623 | 0.625 | 0.662 | 0.651 | 0.625 | 0.623 | 0.642 | 0.651 | 0.681 | 0.670 | 0.661 | 0.662 | 1.000 |  |  |  |  |  |  |  |  |  |  |  |  |  |
| 14 | 0.649 | 0.662 | 0.688 | 0.642 | 0.714 | 0.670 | 0.648 | 0.700 | 0.681 | 0.647 | 0.710 | 0.761 | 0.674 | 1.000 |  |  |  |  |  |  |  |  |  |  |  |  |
| 15 | 0.651 | 0.649 | 0.664 | 0.649 | 0.713 | 0.662 | 0.632 | 0.719 | 0.703 | 0.660 | 0.801 | 0.724 | 0.658 | 0.733 | 1.000 |  |  |  |  |  |  |  |  |  |  |  |
| 16 | 0.610 | 0.629 | 0.687 | 0.684 | 0.661 | 0.660 | 0.664 | 0.661 | 0.703 | 0.677 | 0.688 | 0.664 | 0.742 | 0.693 | 0.697 | 1.000 |  |  |  |  |  |  |  |  |  |  |
| 17 | 0.670 | 0.680 | 0.677 | 0.645 | 0.674 | 0.722 | 0.651 | 0.674 | 0.681 | 0.664 | 0.696 | 0.709 | 0.651 | 0.723 | 0.719 | 0.710 | 1.000 |  |  |  |  |  |  |  |  |  |
| 18 | 0.595 | 0.602 | 0.622 | 0.610 | 0.636 | 0.618 | 0.622 | 0.631 | 0.652 | 0.647 | 0.664 | 0.668 | 0.639 | 0.657 | 0.664 | 0.690 | 0.662 | 1.000 |  |  |  |  |  |  |  |  |
| 19 | 0.632 | 0.654 | 0.636 | 0.613 | 0.775 | 0.649 | 0.631 | 0.671 | 0.647 | 0.644 | 0.707 | 0.709 | 0.642 | 0.737 | 0.759 | 0.678 | 0.703 | 0.671 | 1.000 |  |  |  |  |  |  |  |
| 20 | 0.660 | 0.649 | 0.658 | 0.638 | 0.641 | 0.671 | 0.765 | 0.670 | 0.726 | 0.642 | 0.662 | 0.652 | 0.655 | 0.675 | 0.694 | 0.685 | 0.696 | 0.658 | 0.687 | 1.000 |  |  |  |  |  |  |
| 21 | 0.684 | 0.680 | 0.671 | 0.639 | 0.694 | 0.667 | 0.648 | 0.743 | 0.661 | 0.644 | 0.672 | 0.685 | 0.634 | 0.732 | 0.748 | 0.667 | 0.729 | 0.674 | 0.743 | 0.739 | 1.000 |  |  |  |  |  |
| 22 | 0.671 | 0.652 | 0.675 | 0.649 | 0.632 | 0.654 | 0.658 | 0.681 | 0.752 | 0.619 | 0.665 | 0.632 | 0.647 | 0.649 | 0.677 | 0.677 | 0.655 | 0.664 | 0.652 | 0.732 | 0.716 | 1.000 |  |  |  |  |
| 23 | 0.616 | 0.632 | 0.629 | 0.641 | 0.632 | 0.648 | 0.644 | 0.655 | 0.662 | 0.735 | 0.651 | 0.635 | 0.644 | 0.647 | 0.674 | 0.662 | 0.655 | 0.612 | 0.647 | 0.732 | 0.727 | 0.740 | 1.000 |  |  |  |
| 24 | 0.610 | 0.621 | 0.675 | 0.644 | 0.649 | 0.668 | 0.632 | 0.678 | 0.674 | 0.639 | 0.662 | 0.644 | 0.629 | 0.675 | 0.685 | 0.674 | 0.672 | 0.649 | 0.655 | 0.720 | 0.719 | 0.752 | 0.763 | 1.000 |  |  |
| 25 | 0.632 | 0.636 | 0.634 | 0.639 | 0.636 | 0.615 | 0.628 | 0.636 | 0.644 | 0.635 | 0.632 | 0.608 | 0.737 | 0.657 | 0.638 | 0.716 | 0.642 | 0.674 | 0.648 | 0.681 | 0.688 | 0.707 | 0.701 | 0.727 | 1.000 |  |
| 26 | 0.648 | 0.652 | 0.641 | 0.635 | 0.765 | 0.645 | 0.621 | 0.670 | 0.642 | 0.636 | 0.674 | 0.690 | 0.623 | 0.713 | 0.726 | 0.657 | 0.690 | 0.655 | 0.785 | 0.674 | 0.762 | 0.671 | 0.685 | 0.709 | 0.704 | 1.000 |

Table S7 Molecular variance analysis (AMOVA) of *I. germanica*

| Source of variation | Df | Sum of squares | Mean Squares | Percentage of variation/% |
| --- | --- | --- | --- | --- |
| Three groups (Beijing group, Hebei group, Other group) | | | | |
| Among populations | 2 | 396.37 | 198.19 | 18.32 |
| Within populations | 23 | 1767.65 | 76.85 | 81.68 |

Table S8 Description of morphological characters recorded in *Iris*

| Morphological characters | Abbreviation | Description |
| --- | --- | --- |
| Leaf length 1 | L1 | Length of the longer leaf in the second pair of expanded leaves |
| Leaf width 1 | W1 | Width at the widest part of the longer leaf in the second pair of expanded leaves |
| Leaf length 1/ Leaf width 1 | L1/W1 | Length-to-width ratio of the longer leaf in the second pair of expanded leaves |
| Leaf length 2 | L2 | Length of the shorter leaf in the second pair of expanded leaves |
| Leaf width 2 | W2 | Width at the widest part of the shorter leaf in the second pair of expanded leaves |
| Leaf length 2/ Leaf width 2 | L2/W2 | Length-to-width ratio of the shorter leaf in the second pair of expanded leaves |
| Scape height | SH | Distance from the ground to the lower end of the peduncle |
| Flower height | FH | Distance from the lower end of the drooping petal to the upper end of the standard petal |
| Flower diameter | FD | Maximum diameter of the flower |
| Peduncle length | PL | Distance from the lower end of the drooping petal to the upper end of the scape |
| Width of standard petal | SW | Width at the widest part of the standard petal |
| Width of drooping petal | DW | Width at the widest part of the drooping petal |
| Length of standard petal | SL | Length from the upper end to the lower end of the standard petal |
| Length of drooping petal | DL | Length from the upper end to the lower end of the drooping petal |
| Length of fruit | FrL | Length from the upper end to the lower end of the fruit |
| Width of fruit | FrW | Width at the widest part of the fruit |
| Length of seed | SeL | Length from the upper end to the lower end of the seed |
| Width of seed | SeW | Width at the widest part of the seed |
| Length of anther | AL | Length from the upper end to the lower end of the anther |
| Width of anther | AW | Width at the widest part of the anther |
| Length of perianth tube | PL | Length from the upper end to the lower end of the perianth tube |
| Width of perianth tube | PW | Width at the widest part of the perianth tube |
| Ovary length at flowering | OL | Length from the upper end to the lower end of the ovary |
| Ovary width at flowering | OW | Width at the widest part of the ovary |
| Length of style branch | StL | Length from the upper end to the lower end of the style branch |
| Width of style branch | StW | Width at the widest part of the style branch |
| Scape diameter | SD | Maximum diameter of the scape |

Table S9 The mean value and standard deviation of morphological traits of 20 populations in *Iris*

| Number | L1 | W1 | L1/W1 | L2 | W2 | L2/W2 | SH | FH | FD | PL | SW | DW | SL | DL |
| --- | --- | --- | --- | --- | --- | --- | --- | --- | --- | --- | --- | --- | --- | --- |
| 1 | 35.50±6.70 | 2.59±0.34 | 13.70±2.17 | 31.86±9.02 | 2.63±0.35 | 11.96±2.08 | 50.22±3.10 | 7.63±1.85 | 9.54±1.25 | 3.06±0.78 | 3.78±0.28 | 3.71±0.43 | 7.09±0.48 | 6.58±0.73 |
| 2 | 24.96±2.04 | 3.33±0.61 | 7.59±0.74 | 20.20±3.37 | 2.83±0.40 | 7.12±0.45 | 45.34±4.30 | 6.63±0.09 | 9.91±1.21 | 3.20±0.71 | 3.98±0.37 | 4.19±0.36 | 6.61±0.65 | 6.74±0.55 |
| 3 | 30.82±2.53 | 3.85±0.75 | 8.26±2.01 | 24.76±3.84 | 3.73±0.41 | 6.77±1.85 | 41.44±3.71 | 6.12±1.64 | 12.22±0.97 | 4.87±0.23 | 4.79±0.25 | 5.04±0.28 | 7.28±0.18 | 7.53±0.47 |
| 4 | 30.90±5.05 | 3.59±0.17 | 8.59±1.11 | 25.81±5.54 | 3.39±0.34 | 7.66±1.74 | 49.49±5.19 | 10.70±1.02 | 11.34±1.42 | 4.80±0.51 | 5.54±0.45 | 4.94±0.60 | 8.28±0.76 | 8.32±0.84 |
| 5 | 23.59±4.39 | 1.96±0.13 | 11.96±1.47 | 16.63±4.07 | 1.72±0.13 | 9.65±2.23 | 16.07±1.76 | 5.24±0.30 | 8.43±1.34 | 5.84±0.31 | 3.31±0.43 | 3.07±0.50 | 5.47±0.94 | 5.63±0.72 |
| 6 | 37.08±7.43 | 3.34±0.29 | 11.07±1.67 | 28.75±8.73 | 2.70±0.50 | 10.52±1.64 | 62.99±9.78 | 8.23±0.55 | 10.79±0.87 | 3.51±0.28 | 5.20±0.18 | 4.68±0.28 | 6.92±0.45 | 7.03±0.30 |
| 7 | 16.59±2.13 | 1.69±0.09 | 9.81±0.73 | 13.54±2.75 | 1.71±0.10 | 7.89±1.22 | 10.68±2.72 | 6.24±0.09 | 9.49±1.53 | 5.17±0.80 | 3.67±0.63 | 3.57±0.67 | 6.46±0.87 | 6.57±1.01 |
| 8 | 38.64±5.27 | 3.77±0.33 | 10.27±1.32 | 28.99±8.29 | 3.43±0.22 | 8.39±1.95 | 46.69±3.54 | 10.12±0.17 | 10.86±1.39 | 4.08±0.22 | 5.28±0.60 | 4.82±0.78 | 7.60±0.91 | 7.51±0.96 |
| 9 | 14.94±6.13 | 1.92±0.08 | 7.81±3.29 | 10.70±7.07 | 1.59±0.16 | 6.68±4.26 | 10.21±2.87 | 6.12±0.17 | 9.86±1.02 | 4.92±0.83 | 3.55±0.58 | 3.65±0.56 | 6.78±1.18 | 6.84±0.82 |
| 10 | 39.23±3.41 | 3.12±0.09 | 12.60±1.43 | 35.95±0.57 | 3.40±0.20 | 10.62±0.79 | 38.93±1.03 | 10.68±0.83 | 11.59±0.88 | 4.72±0.21 | 4.83±0.07 | 5.26±0.11 | 7.73±0.16 | 7.88±0.10 |
| 11 | 27.91±9.19 | 2.12±0.23 | 13.03±3.75 | 21.88±9.09 | 1.96±0.23 | 11.15±4.56 | 16.63±2.85 | 6.03±0.53 | 10.46±1.61 | 5.19±1.49 | 3.96±0.42 | 3.59±0.34 | 6.35±0.91 | 6.06±0.65 |
| 12 | 36.25±10.66 | 2.66±0.12 | 13.72±4.30 | 21.35±8.63 | 2.17±0.16 | 9.68±3.30 | 43.66±4.00 | 10.72±0.51 | 14.81±2.20 | 4.48±0.57 | 6.26±0.76 | 6.90±1.10 | 8.51±0.77 | 8.96±1.06 |
| 13 | 36.91±4.34 | 3.36±0.26 | 11.07±1.96 | 28.71±5.15 | 2.74±0.10 | 10.46±1.80 | 56.12±9.31 | 8.10±0.68 | 11.70±2.22 | 4.72±0.33 | 5.35±0.64 | 5.29±0.51 | 7.56±0.92 | 7.60±0.44 |
| 14 | 41.99±12.38 | 4.05±0.33 | 10.26±2.23 | 34.43±18.56 | 3.37±0.68 | 9.88±3.86 | 57.81±5.94 | 10.72±1.81 | 11.06±1.61 | 4.78±0.33 | 5.87±0.67 | 5.81±0.59 | 8.26±0.93 | 8.63±0.48 |
| 15 | 18.23±5.61 | 2.16±0.10 | 8.53±3.04 | 14.90±5.76 | 2.27±0.23 | 6.45±1.86 | 13.15±2.88 | 7.97±0.89 | 11.21±2.93 | 6.53±0.89 | 3.68±0.65 | 3.56±0.56 | 6.82±1.24 | 7.20±0.97 |
| 16 | 21.64±4.12 | 1.74±0.06 | 12.53±2.77 | 17.45±3.18 | 1.58±0.16 | 11.02±1.27 | 16.50±2.18 | 4.20±0.49 | 8.51±0.84 | 5.10±0.64 | 3.30±0.28 | 3.46±0.27 | 5.39±0.78 | 5.31±0.58 |
| 17 | 28.07±4.78 | 2.81±0.23 | 9.97±1.14 | 24.42±4.54 | 2.56±0.30 | 9.50±0.85 | 47.09±11.71 | 8.73±1.01 | 9.59±2.02 | 3.17±0.15 | 4.82±0.99 | 4.82±0.60 | 7.13±0.84 | 6.82±0.71 |
| 18 | 30.89±5.17 | 3.20±0.73 | 9.99±2.68 | 27.26±7.26 | 3.03±0.70 | 9.23±2.52 | 46.59±4.09 | 8.79±0.85 | 9.74±1.11 | 4.73±0.65 | 4.47±0.34 | 4.23±0.27 | 7.36±0.51 | 7.64±0.66 |
| 19 | 37.80±2.30 | 3.93±0.52 | 9.76±1.71 | 35.52±3.32 | 3.85±0.41 | 9.35±1.72 | 52.62±17.58 | 9.62±1.92 | 13.13±4.31 | 4.41±0.50 | 5.58±1.08 | 5.03±1.16 | 8.27±1.53 | 8.05±1.46 |
| 20 | 28.99±4.24 | 1.96±0.13 | 14.82±2.56 | 19.60±2.19 | 1.97±0.15 | 9.97±1.22 | 29.28±2.43 | 7.03±0.46 | 11.24±0.35 | 5.96±0.10 | 4.15±0.19 | 4.14±0.16 | 7.26±0.21 | 7.40±0.34 |

Note: 1: *Iris germanica* ‘Purple Flower’; 2: *Iris germanica* ‘Tawny’; 3: *Iris germanica* ‘Thrilling’; 4: *Iris germanica* ‘Antique Red’; 5: *Iris germanica* ‘Golden Doll’; 6: *Iris germanica* ‘Nautical Flag’; 7: *Iris germanica* ‘Dwarf Dream’; 8: *Iris germanica* ‘Purple Glow’; 9: *Iris germanica* ‘Bloodstone’; 10: *Iris germanica* ‘White Calyx’; 11: *Iris germanica* ‘Sauce Yellow’; 12: *Iris germanica* ‘Immortality’; 13: *Iris germanica* ‘Black Flag’; 14: *Iris germanica* ‘White and Yellow’; 15: *Iris germanica* ‘Cherry Garden’; 16: *Iris germanica* ‘Music Bor’; 17: *Iris germanica* ‘Ussuri’; 18: *Iris germanica* ‘Purple Brown’; 19: *Iris germanica* ‘Indian leader’; 20: *Iris germanica* ‘Flute Sound

Table S9 The mean value and standard deviation of morphological traits of 20 populations in *Iris*

| Number | FrL | FrW | SeL | SeW | AL | AW | PL | PW | OL | OW | StL | StW | SD |
| --- | --- | --- | --- | --- | --- | --- | --- | --- | --- | --- | --- | --- | --- |
| 1 | 5.28±0.09 | 2.27±0.04 | 0.74±0.03 | 0.54±0.03 | 0.98±0.05 | 0.19±0.01 | 1.50±0.18 | 0.33±0.13 | 1.32±0.11 | 0.60±0.19 | 3.38±0.24 | 1.61±0.10 | 0.88±0.08 |
| 2 | 4.50±0.08 | 2.27±0.02 | 0.70±0.07 | 0.42±0.05 | 1.03±0.01 | 0.15±0.05 | 1.75±0.32 | 0.35±0.10 | 1.15±0.23 | 0.46±0.16 | 3.35±0.23 | 1.51±0.01 | 1.12±0.22 |
| 3 | - | - | - | - | 1.47±0.11 | 0.26±0.04 | 2.33±0.12 | 0.59±0.03 | 2.04±0.28 | 0.82±0.04 | 3.37±0.12 | 2.16±0.10 | 1.28±0.17 |
| 4 | 6.17±0.13 | 2.73±0.13 | 0.52±0.03 | 0.30±0.02 | 1.58±0.08 | 0.20±0.11 | 2.10±0.41 | 0.53±0.04 | 1.75±0.15 | 0.71±0.08 | 3.37±0.13 | 1.87±0.24 | 0.94±0.14 |
| 5 | - | - | - | - | 1.41±0.11 | 0.19±0.03 | 3.28±0.73 | 0.31±0.05 | 2.07±0.16 | 0.53±0.12 | 2.77±0.06 | 1.28±0.14 | 0.74±0.07 |
| 6 | 5.23±0.06 | 2.05±0.07 | 0.62±0.04 | 0.50±0.01 | 1.30±0.01 | 0.18±0.01 | 1.57±0.06 | 0.46±0.03 | 1.49±0.18 | 0.65±0.06 | 3.12±0.18 | 2.14±0.06 | 1.03±0.13 |
| 7 | - | - | - | - | 0.87±0.14 | 0.17±0.03 | 3.07±0.27 | 0.43±0.09 | 1.90±0.25 | 0.62±0.11 | 3.07±0.05 | 1.34±0.22 | 0.71±0.06 |
| 8 | - | - | - | - | 1.52±0.12 | 0.18±0.03 | 1.87±0.05 | 0.57±0.07 | 1.67±0.07 | 0.63±0.03 | 3.56±0.37 | 1.88±0.16 | 1.01±0.09 |
| 9 | - | - | - | - | 1.54±0.20 | 0.26±0.06 | 2.93±0.22 | 0.45±0.06 | 1.74±0.35 | 0.60±0.09 | 2.99±0.12 | 1.48±0.09 | 0.73±0.06 |
| 10 | - | - | - | - | 1.64±0.01 | 0.21±0.01 | 1.77±0.02 | 0.56±0.05 | 1.77±0.08 | 0.74±0.01 | 3.88±0.11 | 1.87±0.05 | 0.88±0.05 |
| 11 | - | - | - | - | 1.39±0.17 | 0.23±0.05 | 3.34±0.71 | 0.38±0.02 | 1.96±0.50 | 0.58±0.08 | 3.14±0.33 | 1.67±0.04 | 0.64±0.03 |
| 12 | 8.45±0.09 | 4.25±0.15 | 0.72±0.02 | 0.47±0.03 | 1.07±0.19 | 0.26±0.02 | 1.61±0.23 | 0.84±0.06 | 2.24±0.36 | 1.06±0.17 | 3.93±0.06 | 2.23±0.10 | 1.11±0.02 |
| 13 | 8.15±0.04 | 3.26±0.04 | 0.80±0.01 | 0.48±0.01 | 1.47±0.05 | 0.25±0.04 | 2.01±0.12 | 0.58±0.08 | 2.04±0.32 | 0.92±0.33 | 3.59±0.15 | 1.96±0.02 | 1.02±0.03 |
| 14 | - | - | - | - | 1.33±0.03 | 0.24±0.02 | 1.96±0.17 | 0.64±0.11 | 2.07±0.21 | 0.87±0.18 | 3.84±0.20 | 2.11±0.07 | 1.38±0.43 |
| 15 | - | - | - | - | 1.65±0.14 | 0.21±0.01 | 3.82±0.81 | 0.37±0.03 | 1.81±0.33 | 0.59±0.06 | 3.14±0.38 | 1.39±0.17 | 0.73±0.05 |
| 16 | - | - | - | - | 1.04±0.04 | 0.24±0.06 | 3.28±0.54 | 0.36±0.08 | 1.67±0.41 | 0.44±0.04 | 2.46±0.07 | 1.63±0.12 | 0.58±0.10 |
| 17 | 4.46±0.05 | 2.55±0.04 | 0.57±0.02 | 0.55±0.01 | 1.15±0.14 | 0.19±0.03 | 1.51±0.19 | 0.42±0.01 | 1.29±0.08 | 0.61±0.08 | 3.34±0.31 | 1.93±0.06 | 0.95±0.14 |
| 18 | - | - | - | - | 1.55±0.04 | 0.20±0.01 | 2.61±0.40 | 0.50±0.08 | 1.61±0.27 | 0.67±0.03 | 3.52±0.16 | 1.95±0.16 | 0.79±0.11 |
| 19 | - | - | - | - | 1.68±0.02 | 0.21±0.02 | 2.16±0.25 | 0.52±0.04 | 1.75±0.24 | 0.73±0.14 | 3.43±0.12 | 2.01±0.20 | 1.01±0.16 |
| 20 | - | - | - | - | 1.31±0.02 | 0.26±0.03 | 3.94±0.07 | 0.41±0.02 | 1.70±0.20 | 0.60±0.07 | 3.30±0.13 | 1.50±0.10 | 0.89±0.06 |

Note: 1: *Iris germanica* ‘Purple Flower’; 2: *Iris germanica* ‘Tawny’; 3: *Iris germanica* ‘Thrilling’; 4: *Iris germanica* ‘Antique Red’; 5: *Iris germanica* ‘Golden Doll’; 6: *Iris germanica* ‘Nautical Flag’; 7: *Iris germanica* ‘Dwarf Dream’; 8: *Iris germanica* ‘Purple Glow’; 9: *Iris germanica* ‘Bloodstone’; 10: *Iris germanica* ‘White Calyx’; 11: *Iris germanica* ‘Sauce Yellow’; 12: *Iris germanica* ‘Immortality’; 13: *Iris germanica* ‘Black Flag’; 14: *Iris germanica* ‘White and Yellow’; 15: *Iris germanica* ‘Cherry Garden’; 16: *Iris germanica* ‘Music Bor’; 17: *Iris germanica* ‘Ussuri’; 18: *Iris germanica* ‘Purple Brown’; 19: *Iris germanica* ‘Indian leader’; 20: *Iris germanica* ‘Flute Sound’; “-” indicates missing data.
